# Normative profile of retinal nerve fiber layer thickness using spectral domain optical coherence tomography in healthy Omani adults with emmetropic eyes: a cross-sectional study

**DOI:** 10.1186/s12886-026-05181-1

**Published:** 2026-07-29

**Authors:** Maitha Al Asmi, Sitara Azeem, Hanan Al Harthy, Buthaina Sabt

**Affiliations:** 1 Ophthalmology Residency Training Program, Oman Medical Specialty Board (OMSB), Muscat, Oman; 2 Ophthalmology Department, Sultan Qaboos University Hospital – University Medical City (SQUH-UMC), Muscat, Oman

**Keywords:** Retinal nerve fiber layer, Optical coherence tomography, Oman, Normative database, Healthy adults, Arab population, Thickness, Glaucoma, ISNT pattern, Optic nerve head

## Abstract

**Background:**

Optical coherence tomography (OCT) is essential for diagnosing glaucomatous and neurodegenerative optic neuropathies. However, diagnostic accuracy relies on population-specific normative databases to avoid misinterpretation. As no large-scale reference data exist for the Omani population, this study aimed to establish normative peripapillary retinal nerve fiber layer (pRNFL) thickness values and evaluate the influence of demographic and anatomical factors in healthy Omani adults.

**Methods:**

In this hospital-based cross-sectional study, 378 healthy Omani adults (756 eyes) aged 19–63 years (mean ± SD: 34.30 ± 9.44 years) were recruited. Inclusion criteria included intraocular pressure ≤ 21 mmHg, emmetropic eyes (refractive error between − 1.0 and + 1.0 D), and no ocular or systemic comorbidities. pRNFL thickness and optic nerve head (ONH) parameters were measured using Spectral Domain-OCT (Cirrus HD-OCT 5000) by a single trained technician to ensure data quality. Statistical analysis included independent samples t-tests, one-way ANOVA, and Pearson’s correlation. Reference intervals (RIs) were calculated following CLSI C28-A3 guidelines.

**Results:**

The mean global pRNFL thickness was 95.34 ± 8.83 μm in the right eye (OD) and 95.04 ± 8.65 μm in the left eye (OS). The 95% RI was 79.00–116.00 μm (OD) and 79.47–113.52 μm (OS). Quadrant distribution followed the ISNT rule (Inferior > Superior > Nasal > Temporal). Females demonstrated significantly thicker pRNFL than males (*p* < 0.01). A significant age-related decline in pRNFL thickness was observed (*p* < 0.001), particularly in those ≥ 40 years. pRNFL thickness correlated strongly and positively with neuroretinal rim area (*p* < 0.001) and negatively with average cup-to-disc ratio and cup volume (*p* < 0.05).

**Conclusions:**

This study establishes the first large-scale normative pRNFL database for Omani adults. The findings highlight that age and sex are significant determinants of thickness, emphasizing that population-specific, adjusted reference values are essential for accurate OCT interpretation. Integrating these localized data into clinical practice can reduce diagnostic misclassification, such as “red disease”, and enhance the detection of glaucoma and other optic neuropathies in this population.

## Background

Recent advancements in optical coherence tomography (OCT) have enabled exact quantitative and qualitative assessments of early retinal nerve fiber layer (RNFL) loss through fine structural imaging of the retinal layers [1, 2]. This has made OCT an indispensable tool for the detection, initiation of treatment, and follow-up of a range of ophthalmic and neurodegenerative conditions, particularly glaucoma [1, 2]. In addition, OCT facilitates differentiation between glaucomatous and non-glaucomatous optic nerve cupping, as the pattern of RNFL loss is less diffuse in non-glaucomatous cases [3].

In healthy eyes, peripapillary retinal nerve fiber layer (RNFL) thickness is classically described to follow the ISNT pattern, with the inferior quadrant being the thickest, followed by the superior, nasal, and temporal quadrants [4]. However, this distribution has been shown to vary across different demographic and ethnic populations, underscoring the importance of population-specific evaluation of RNFL patterns.

Despite these advantages, important challenges persist in the interpretation of OCT findings. Although OCT devices rely on normative databases of healthy eyes, which generally ensure precise and reproducible measurements [5, 6], interpretation can be limited by substantial variability across platforms. Differences in imaging technology, software algorithms, and the composition of reference databases contribute to this variability [5, 6]. Individuals from populations underrepresented in these databases are particularly at risk of misinterpretation, as insufficient demographic diversity may compromise diagnostic accuracy [5, 6]. Several studies have highlighted this limitation by demonstrating significant inter-population variation in OCT-derived RNFL measurements, emphasizing the need for population-specific normative datasets [6–8].

For example, a Spectralis spectral-domain OCT (SD-OCT) study reported thicker RNFL values in South African eyes compared with the Spectralis normative database [9]. Similar findings have been demonstrated in China [10], India [11], and Nepal [12]. In Africa, Cirrus HD-OCT 5000 showed that RNFL thickness was significantly greater in Ghanaians compared with other races [8]. Studies using Stratus SD-OCT reported RNFL thickness in Northern Nigerians [13, 14] comparable to Japanese eyes [15] but higher than in Italian populations [16], while intra-ethnic variability has been suggested in Nigeria, where Topcon 3D OCT-1 Maestro measurements revealed thicker RNFL in the south compared with the north [17, 18]. These inter- and intra-population differences highlight the risk of misclassification when relying on non-representative databases. Normative studies have also been conducted in Jordan [19] and Saudi Arabia [20], providing valuable regional insights; however, their relatively small sample sizes indicate a need for larger, more representative datasets. Despite global data, no large-scale normative OCT data exist for the Omani population, which remains underrepresented in current databases. To our knowledge, no studies have yet been performed in Oman. Therefore, this study aimed to establish normative reference values for peripapillary RNFL thickness in healthy adult Omanis using SD-OCT. In addition, it aimed to evaluate the influence of demographic factors (age and sex) and anatomical optic nerve head parameters (cup-to-disc ratio (CDR), optic disc area, rim area, and cup volume) on RNFL thickness, and to determine whether the quadrant distribution conforms to the classical ISNT rule.

## Methods

This hospital-based cross-sectional study aimed to establish normative reference values for peripapillary RNFL thickness in healthy adult Omanis. The study was conducted in the Department of Ophthalmology at Sultan Qaboos University Hospital, University Medical City (SQUH-UMC), a tertiary-care teaching center in Muscat, Oman. Ethical approval was obtained from the Medical Research Ethics Committee at Sultan Qaboos University (REF. NO. SQU-EC/ 067\2023. MREC #3097). Informed consent was taken from the participants, then a checklist was used to confirm that participants had no ocular and/or medical comorbidities. Data such as date of birth and sex were gathered from the participants. Auto-refraction was performed for each participant to identify any refractive errors. Intraocular pressure (IOP) was checked using the iCare rebound tonometer (iCare Finland Oy, Vantaa, Finland). All participants underwent slit lamp examination to screen for any anterior or posterior segment abnormalities. Other parameters, such as RNFL thickness (average, inferior, superior, nasal, and temporal), average cup-to-disc ratio (CDR), optic disc area, rim area & cup volume were collected from Optical Coherence Tomography (OCT) results using the Cirrus HD-OCT 5000 (Carl Zeiss Meditec, Dublin, CA, USA). The Optic Disc Cube 200 × 200 scan protocol, centered on the optic nerve head, was used for RNFL analysis. The data were then entered into a data collection sheet on Microsoft Excel (Microsoft Corp., Redmond, WA, USA).

Omani adults aged 18 years and above were recruited using convenience sampling from individuals present in the department, including patients, visitors, attendants, staff, and trainees. Participants were excluded if they had a refractive error outside the range of − 1.0 to + 1.0 D (sphere or cylinder), an intraocular pressure (IOP) greater than 21 mmHg, any abnormalities on anterior or posterior segment screening, any known anterior segment or retinal disease, a history of amblyopia, previous ocular surgery, or any other medical comorbidities such as diabetes or hypertension.

As per the CLSI C28-A3 guideline, the percentile method is recommended for sample sizes above 120 to calculate 90% confidence intervals for the reference limits. To obtain more reliable reference limits with narrower 90% confidence intervals, a larger sample size is required. Given the well-established negative correlation between age and RNFL thickness, the sample was stratified into three age groups: 18–29 years, 30–39 years, and ≥ 40 years. Accordingly, we aimed to recruit at least 120 healthy subjects in each group.

### Data quality

To minimize inter-observer variability, all high-resolution OCT images (signal strength ≥ 6/10) were acquired by a single technician, while participant screening and data entry were performed exclusively by a single specialist and a single investigator, respectively.

### Statistical analysis

Continuous data were presented as the mean, median, and standard deviation, whereas categorical data were presented as frequency and percentage. A 95% two-sided reference interval was reported for the normal RNFL thickness, with a 90% confidence interval for both the lower and upper limits of the reference interval. The comparison of mean scores between two independent groups was performed using the independent samples t-test, while the comparison of mean scores between three independent groups was performed using One-way ANOVA. Correlation between two continuous variables was assessed using Pearson’s correlation coefficient. A p-value less than 0.05 was considered statistically significant. The reference interval was calculated using MedCalc software, version 23 (MedCalc Software Ltd, Ostend, Belgium), and the remaining statistical analyses were performed in IBM SPSS Statistics, version 30.0 (IBM Corp., Armonk, NY, USA).

## Results

Data from 756 eyes (378 patients) were analyzed. Their ages ranged between 19 and 63 years with a mean ± SD of 34.30 ± 9.44 years. There were 163 (43.1%) males and 215 (56.9%) females. For the following age groups: 18–29, 30–39, and ≥ 40 years, there were 131 (34.7%), 127 (33.6%), and 120 (31.7%) participants, respectively.

The mean average retinal nerve fiber layer (RNFL) thickness overall was 95.34 ± 8.83 μm in the right eye (OD) and 95.04 ± 8.65 μm in the left eye (OS), as presented in Tables 1 and 2. The 95% reference interval for the overall average RNFL thickness ranged from 79.00 to 116.00 μm in OD and from 79.47 to 113.52 μm in OS, with 90% confidence intervals provided for both the lower and upper limits (Table 3).

**Table 1:** Overall descriptive statistics for the right eye (OD)

		AVG RNFL Thickness	RNFL I	RNFL S	RNFL *N*	RNFL T	AVG CDR	Disc Area	Rim Area	Cup Volume
*n*	Valid	378	378	378	378	378	378	378	378	378
	Missing	0	0	0	0	0	0	0	0	0
Mean		95.34	124.33	117.59	73.30	65.83	0.47	1.85	1.36	0.16
Median		95.00	123.00	117.00	71.00	65.00	0.49	1.81	1.33	0.11
Std. Deviation		8.83	15.42	16.00	11.10	10.22	0.15	0.35	0.22	0.17
Minimum		73.00	74.00	11.00	53.00	42.00	0.06	0.53	0.87	0.00
Maximum		123.00	179.00	162.00	144.00	146.00	0.79	3.24	2.02	1.28
Percentiles	Q1	89.00	113.00	107.75	66.00	59.00	0.39	1.61	1.20	0.05
	Q2	95.00	123.00	117.00	71.00	65.00	0.49	1.81	1.33	0.11
	Q3	102.00	134.00	128.00	80.00	72.00	0.59	2.05	1.50	0.21

Average (AVG); Retinal Nerve Fiber Layer (RNFL); Inferior (I); Superior (S), Nasal (N); Temporal (T); Cup-to-Disc Ratio (CDR)

**Table 2:** Overall descriptive statistics for the left eye (OS)

		AVG RNFL Thickness	RNFL I	RNFL S	RNFL *N*	RNFL T	AVG CDR	Disc Area	Rim Area	Cup Volume
*n*	Valid	378	378	378	378	378	377	378	376	378
	Missing	0	0	0	0	0	1	0	2	0
Mean		95.04	125.85	119.94	71.15	63.55	0.47	1.87	1.38	0.15
Median		95.00	125.00	120.00	70.00	64.00	0.50	1.82	1.34	0.11
Std. Deviation		8.65	15.55	15.06	10.99	8.35	0.16	0.34	0.23	0.15
Minimum		73.00	94.00	49.00	49.00	42.00	0.06	0.27	0.93	0.00
Maximum		121.00	178.00	165.00	122.00	88.00	0.78	3.02	2.29	1.08
Percentiles	Q1	89.00	114.00	109.00	63.00	58.00	0.36	1.62	1.20	0.04
	Q2	95.00	125.00	120.00	70.00	64.00	0.50	1.82	1.34	0.11
	Q3	101.00	136.00	129.00	78.00	69.25	0.59	2.08	1.52	0.21

Average (AVG); Retinal Nerve Fiber Layer (RNFL); Inferior (I); Superior (S), Nasal (N); Temporal (T); Cup-to-Disc Ratio (CDR)

**Table 3:** Reference intervals for the average RNFL thickness for the right (OD) and left (OS) eyes

Average RNFL Thickness (µm)	OD		OS	
	95% Reference Interval (Double-sided)		95% Reference Interval (Double-sided)	
	Lower limit (90% CI)	Upper limit (90% CI)	Lower limit (90% CI)	Upper limit (90% CI)
Overall	79.00 (78.00–82.00)	116.00 (111.00-119.00)	79.47 (78.00–81.00)	113.52 (111.00-116.00)
Sex				
Male	76.01 (73.99–78.02)	111.31 (109.29-113.32)	78.10 (73.00–80.00)	113.70 (107.00-119.00)
Female	79.97 (78.31–81.62)	113.24 (111.59–114.90)	82.40 (79.00–83.00)	113.60 (111.00-120.00)
Age				
18–29 years	79.44 (77.21–81.67)	114.43 (112.20-116.65)	80.11 (78.01–82.21)	113.09 (110.99-115.19)
30–39 years	79.42 (77.22–81.63)	113.61 (111.41-115.81)	78.44 (76.15–80.73)	114.02 (111.73-116.31)
40 years and above	78.02 (73.00–79.00)	107.00 (106.00-118.00)	77.05 (75.04–79.05)	107.14 (105.14-109.15)

Retinal nerve fiber layer (RNFL); Confidence Interval (CI)

Although statistically significant inter-eye differences were observed in sectoral RNFL thickness (inferior, superior, nasal, and temporal), rim area, and cup volume (*p* < 0.05), the mean differences were small (ranging from − 2.35 to + 2.28), indicating minimal clinical significance. No significant differences were found in average RNFL thickness, average CDR, or disc area (*p* > 0.05), suggesting overall symmetry between eyes.

Quadrant-wise analysis (Tables 1 and 2) demonstrated that the inferior quadrant showed the greatest thickness (OD: 124.33 ± 15.42 μm; OS: 125.85 ± 15.55 μm), followed by the superior (OD: 117.59 ± 16.00 μm; OS: 119.94 ± 15.06 μm), nasal (OD: 73.30 ± 11.10 μm; OS: 71.15 ± 10.99 μm), and temporal quadrants (OD: 65.83 ± 10.22 μm; OS: 63.55 ± 8.35 μm), respectively. This distribution is consistent with the classical ISNT rule (Inferior > Superior > Nasal > Temporal) described in healthy populations worldwide.

Analysis revealed statistically significant sex-specific differences in RNFL thickness. Females demonstrated greater mean average RNFL thickness compared to males in both eyes (OD: 96.61 ± 8.49 μm vs. 93.66 ± 9.00 μm, *p* = 0.001; OS: 96.20 ± 8.29 μm vs. 93.51 ± 8.89 μm, *p* = 0.003). Quadrant-wise, females exhibited significantly thicker RNFLs in the inferior and temporal quadrants of both eyes. Additionally, females had significantly smaller average cup-to-disc ratios and cup volumes compared with males, corresponding to relatively larger rim areas (Tables 4 and 5).

**Table 4:** Optic nerve head (ONH) parameters of the right eye (OD) by age and sex

Variable	*n* (%)	AVG RNFL Thickness	RNFL I	RNFL S	RNFL *N*	RNFL T	AVG CDR	Disc Area	Rim Area	Cup Volume
		Mean ± SD	Mean ± SD	Mean ± SD	Mean ± SD	Mean ± SD	Mean ± SD	Mean ± SD	Mean ± SD	Mean ± SD
Sex										
Male	163 (43.1)	93.66 ± 9.00	121.53 ± 15.44	116.69 ± 15.14	72.26 ± 10.16	64.61 ± 10.53	0.51 ± 0.14	1.89 ± 0.39	1.34 ± 0.22	0.19 ± 0.18
Female	215 (56.9)	96.61 ± 8.49	126.44 ± 15.11	118.28 ± 16.62	74.08 ± 11.72	66.76 ± 9.90	0.45 ± 0.16	1.82 ± 0.32	1.38 ± 0.22	0.14 ± 0.15
P-value		0.001*	0.002*	0.339	0.115	0.043*	< 0.001*	0.030*	0.051	0.002*
Age										
18–29 years	131 (34.7)	96.94 ± 8.93	126.27 ± 16.40	119.67 ± 17.87	74.77 ± 12.72	65.87 ± 9.18	0.44 ± 0.16	1.87 ± 0.34	1.42 ± 0.23	0.15 ± 0.18
30–39 years	127 (33.6)	96.46 ± 8.73	125.90 ± 15.05	118.61 ± 15.15	73.96 ± 10.75	67.12 ± 8.74	0.46 ± 0.15	1.82 ± 0.30	1.37 ± 0.22	0.13 ± 0.13
>=40 years	120 (31.7)	92.40 ± 8.13	120.53 ± 14.10	114.25 ± 14.20	70.99 ± 9.10	64.43 ± 12.41	0.52 ± 0.13	1.86 ± 0.41	1.28 ± 0.19	0.20 ± 0.18
P-value		< 0.001*	0.005*	0.018*	0.018*	0.119	< 0.001*	0.493	< 0.001*	0.003*

*Statistically significant, Test: Independent samples t-test, One-way ANOVA

Average (AVG); Retinal nerve fiber layer (RNFL); Inferior (I); Superior (S), Nasal (N); Temporal (T); Cup-to-Disc Ratio (CDR); Standard Deviation (SD)

**Table 5:** Optic nerve head (ONH) parameters of the left eye (OS) by age and sex

Variable	*n* (%)		AVG RNFL Thickness		RNFL I		RNFL S		RNFL *N*		RNFL T		AVG CDR		Disc Area		Rim Area		Cup Volume	
			Mean ± SD		Mean ± SD		Mean ± SD		Mean ± SD		Mean ± SD		Mean ± SD		Mean ± SD		Mean ± SD		Mean ± SD	
Sex																				
Male	163 (43.1)		93.51 ± 8.89		123.58 ± 16.01		118.31 ± 14.99		70.44 ± 10.51		62.21 ± 7.90		0.50 ± 0.15		1.90 ± 0.39		1.35 ± 0.23		0.17 ± 0.17	
Female	215 (56.9)		96.20 ± 8.29		127.57 ± 15.01		121.18 ± 15.03		71.70 ± 11.34		64.56 ± 8.56		0.44 ± 0.16		1.84 ± 0.31		1.40 ± 0.23		0.13 ± 0.13	
P-value		0.003*		0.013*		0.066		0.269		0.007*		< 0.001*		0.149		0.037*		0.004*		
Age																				
18–29 years	131 (34.7)		96.60 ± 8.41		127.93 ± 15.59		123.02 ± 14.26		72.65 ± 10.80		63.69 ± 7.67		0.43 ± 0.17		1.86 ± 0.36		1.44 ± 0.24		0.13 ± 0.14	
30–39 years	127 (33.6)		96.20 ± 9.11		127.39 ± 15.98		121.12 ± 14.38		71.49 ± 11.59		64.76 ± 8.48		0.45 ± 0.16		1.84 ± 0.30		1.39 ± 0.23		0.13 ± 0.12	
>=40 years	120 (31.7)		92.10 ± 7.68		121.96 ± 14.43		115.33 ± 15.62		69.17 ± 10.32		62.12 ± 8.76		0.52 ± 0.14		1.90 ± 0.37		1.29 ± 0.19		0.19 ± 0.17	
P-value		< 0.001*		0.004*		< 0.001*		0.039*		0.044*		< 0.001*		0.426		< 0.001*		< 0.001*		

*Statistically significant, Test: Independent samples t-test, One-way ANOVA

Average (AVG); Retinal nerve fiber layer (RNFL); Inferior (I); Superior (S), Nasal (N); Temporal (T); Cup-to-Disc Ratio (CDR); Standard Deviation (SD)

RNFL thickness demonstrated a significant age-related decline. The mean average RNFL thickness was highest in the 18–29 years group (OD: 96.94 ± 8.93 μm; OS: 96.60 ± 8.41 μm), slightly lower in the 30–39 years group (OD: 96.46 ± 8.73 μm; OS: 96.20 ± 9.11 μm), and lowest in participants ≥ 40 years (OD: 92.40 ± 8.13 μm; OS: 92.10 ± 7.68 μm) (*p* < 0.001). Quadrant-wise analysis revealed significantly reduced inferior and superior RNFL thicknesses in the ≥ 40 years group compared to younger participants. Advancing age was also associated with larger average cup-to-disc ratios and cup volumes, alongside significantly smaller rim areas in both eyes (Tables 4 and 5).

Correlation analysis demonstrated strong and statistically significant associations between average RNFL thickness and optic nerve head (ONH) parameters in both eyes. Average RNFL thickness showed strong positive correlations with inferior and superior quadrant RNFL thicknesses (*r* = 0.729–0.855, *p* < 0.001), and moderate positive correlations with rim area (*r* = 0.454–0.509, *p* < 0.001). Conversely, significant negative correlations were observed with average cup-to-disc ratio (*r* = − 0.266 to − 0.268, *p* < 0.001) and cup volume (*r* = − 0.102 to − 0.143, *p* < 0.05). Disc area demonstrated a weak positive correlation with RNFL thickness (*r* = 0.154–0.169, *p* ≤ 0.003). These findings were consistent between right and left eyes.

## Discussion

The present study establishes the first large spectral-domain OCT (SD-OCT) normative dataset for peripapillary retinal nerve fiber layer (pRNFL) thickness in an adult Omani cohort (*n* = 756 eyes). The mean global RNFL thickness (OD: 95.34 ± 8.83 μm; OS: 95.04 ± 8.65 μm) lies within the intermediate range of previously reported normative values [15, 21, 22]. When compared across populations using similar SD-OCT platforms, Omani values show both concordance and systematic ethnic variation. Our findings closely mirror those of multiethnic Asian cohorts from the Singapore Epidemiology of Eye Diseases Study, aligning with Chinese (95.7 μm) and Malay (94.9 μm) participants, while remaining significantly thicker than values reported in Indian populations (~ 87.3 μm) [23].

Regionally, Omani RNFL thickness appears slightly thinner than that reported in Jordanian adults (99 ± 11 μm) [19]. In contrast, East Asian and African cohorts generally demonstrate thicker measurements; Japanese populations report mean values ranging from 103.5 to 111.8 μm [15, 24], while Southern Nigerian cohorts average approximately 111.5 μm [17]. Large multiethnic Western datasets report values comparable to or slightly higher than ours, including the study by Alasil et al. (97.3 ± 9.6 μm) and Budenz et al. (~ 100 μm) [4, 21]. These inter-population differences of 5–15 μm, consistently observed even when using identical OCT platforms, underscore ethnicity as a major determinant of RNFL thickness [21, 22]. Although direct statistical comparisons across ethnic groups or with manufacturer-provided normative databases were not performed, our findings were interpreted in the context of published literature, which indicates that these databases are predominantly derived from Caucasian populations and may not fully represent other ethnic groups. The consistency of these differences across studies using similar SD-OCT platforms suggests that the observed variation is unlikely to be solely attributable to device-related factors, thereby supporting the need for population-specific normative databases. Importantly, reliance on manufacturer-provided normative databases, which are predominantly derived from Caucasian populations with reported means as low as 93 μm, may increase the risk of misclassification, particularly for “red disease,” when applied to Omani patients [4–7, 21].

Quadrant analysis in our cohort confirmed the classical ISNT pattern (Inferior > Superior > Nasal > Temporal), with the inferior quadrant demonstrating the greatest thickness [11]. This configuration is consistent with findings from large normative studies and pediatric and adult populations worldwide [4, 10, 11, 24, 25]. Preservation of this pattern supports the internal validity of the Omani dataset. However, it is important to note that the inclusion of only emmetropic eyes in this study may have minimized the influence of refractive error on RNFL distribution, which is known to alter quadrant thickness profiles. Therefore, the observed adherence to the ISNT rule may, in part, reflect this selection criterion rather than ethnicity alone. Despite ethnic variation in absolute thickness values, the fundamental topographic arrangement of nerve fiber convergence appears to remain conserved [18]. Notably, this differs from findings in some Middle Eastern and African populations, such as the Jordanian cohort where the ISNT rule was absent in the majority of eyes, and Northern Nigerian and Japanese studies where the superior quadrant often predominates [13–15, 19].

A statistically significant age-related decline in pRNFL thickness was observed, with participants aged ≥ 40 years demonstrating lower values compared with younger groups [13]. The magnitude of decline in our cohort aligns with the widely reported physiological thinning of approximately 2–5 μm per decade [18, 21]. Specifically, our findings are comparable to the 2.0 μm per decade reported by Budenz et al. [21], the 2.26 μm per decade described in Nepalese populations [12], and similar estimates from Japanese cohorts [24]. These results reinforce the clinical necessity of age-adjusted reference intervals to prevent misinterpretation of normal aging as glaucomatous damage, particularly in older adults, while also improving sensitivity for early disease detection in younger individuals [18, 24].

Sex-related differences were also observed, with Omani females demonstrating slightly thicker global RNFL measurements than males (mean difference ~ 2–3 μm) [12, 19]. This finding is consistent with reports from Northern Nigerian and Chinese pediatric cohorts, which similarly documented thicker RNFL values in females [14, 25]. However, other large-scale studies from the United States and Singapore found no significant sex effect after adjusting for disc area and axial length [4, 21, 23]. The modest difference observed in our cohort likely reflects underlying anatomical factors rather than true biological disparity. Females generally have shorter axial lengths, leading to less stretching of nerve fibers and consequently thicker RNFL measurements [26]. Additionally, fixed-diameter scan circles may sample tissue further from the disc margin in larger optic discs, more commonly seen in males, resulting in relatively thinner apparent measurements [27]. Given the small effect size, these differences are unlikely to exceed inter-scan variability at the individual level and therefore have limited standalone clinical significance [10, 11].

Consistent with this structural basis, correlation analysis revealed a strong relationship between RNFL thickness and optic nerve head (ONH) parameters. RNFL thickness correlated positively with neuroretinal rim area and negatively with cup-to-disc ratio (CDR) [14, 20]. These findings align with results from the Handan Eye Study and other large normative datasets, confirming that eyes with larger rims naturally contain more axons, while those with larger cups demonstrate thinner RNFL [10, 11, 21, 22]. Disc area also exerted a notable influence on RNFL measurements; larger discs often yielded thicker values because the standard 3.4-mm scanning circle samples tissue closer to the disc margin, where fibers are denser [27]. These observations highlight the importance of interpreting RNFL measurements in conjunction with ONH morphology rather than in isolation.

Taken together, these findings emphasize the combined influence of biological, demographic, and methodological factors on RNFL thickness. Age, sex, disc size, and ethnicity all contribute to normal variability, while OCT platform differences introduce additional systematic variation. Within this context, the provision of Omani-specific normative data represents a critical step toward improving diagnostic accuracy. Integration of these localized reference intervals into clinical practice has the potential to enhance both sensitivity and specificity for glaucoma detection, particularly in a region characterized by significant ethnic heterogeneity [5, 6, 19, 21, 24].

The strengths of this study include its large sample size, use of high-resolution SD-OCT imaging, and comprehensive analysis incorporating quadrant distribution and ONH correlations. However, several limitations should be acknowledged. As a single-center, hospital-based study using convenience sampling, the findings may not fully represent the genetic and ethnic diversity of the broader Omani population. Ethnic subgroup analysis within Oman was not performed, and the absence of axial length, refractive error, and visual field data may have influenced RNFL estimates. Additionally, the inclusion of only emmetropic eyes further limits the generalizability of these findings and may have influenced the observed RNFL distribution pattern, including preservation of the ISNT rule. Future multicenter studies incorporating detailed ocular biometry, functional testing, and longitudinal follow-up would further refine these reference standards.

In summary, this study establishes population-specific normative values for pRNFL thickness in Omani adults. Incorporation of these data into OCT interpretation frameworks may reduce diagnostic misclassification, support earlier detection of glaucomatous damage, and provide a reliable reference for both clinical care and future research in this population.

## Conclusion

This study establishes the first large-scale normative database for peripapillary retinal nerve fiber layer (pRNFL) thickness in a healthy adult Omani cohort using spectral-domain OCT. Our findings demonstrate a mean global RNFL thickness of approximately 95 μm, confirming that the Omani population occupies an intermediate structural position compared to East Asian and Caucasian cohorts. The preservation of the classical ISNT pattern in this cohort supports the internal validity of the dataset and provides a reliable topographic benchmark for clinical assessment.

A critical finding of this research is that age and sex are significant independent determinants of RNFL thickness in Omanis, with a physiological decline of approximately 2 μm per decade and significantly thicker global measurements in females. These results emphasize that the reliance on generic, predominantly Caucasian manufacturer databases may lead to diagnostic inaccuracies, such as the misclassification of physiological age-related thinning as “red disease” or pathological thinning.

The key conclusion of this study is that population-specific, age- and sex-adjusted normative data are essential for the accurate interpretation of OCT scans in the diagnosis and monitoring of glaucoma and other optic neuropathies. Integrating Omani-specific reference values into clinical practice can significantly refine diagnostic accuracy, reduce misclassification of physiological age-related thinning, and promote earlier recognition of disease. This work addresses a critical gap by providing locally relevant OCT data that will strengthen ophthalmic practice in Oman and serve as a necessary foundation for future multicenter and longitudinal studies in this unique and heterogeneous population.

## Data Availability

The datasets used and/or analyzed during the current study are available from the corresponding author on reasonable request.

## Abbreviations

ANOVA: One-way Analysis of Variance
CDR: Cup-to-Disc Ratio
CLSI: Clinical and Laboratory Standards Institute
IOP: Intraocular Pressure
ISNT: Inferior, Superior, Nasal, and Temporal (quadrant pattern)
OCT: Optical Coherence Tomography
OD: Oculus Dexter (Right eye)
ONH: Optic Nerve Head
OS: Oculus Sinister (Left eye)
pRNFL: Peripapillary Retinal Nerve Fiber Layer
RNFL: Retinal Nerve Fiber Layer
SD-OCT: Spectral-Domain Optical Coherence Tomography
SQUH-UMC: Sultan Qaboos University Hospital – University Medical City
